# Perceived workload and job satisfaction among nurses

**DOI:** 10.1192/j.eurpsy.2025.1873

**Published:** 2025-08-26

**Authors:** A. Abbes, I. Sellami, A. Feki, A. Khelil, M. Hajjaji, S. Baklouti, M. L. Masmoudi, K. Jmal Hammami

**Affiliations:** 1 Occupationnal medecine; 2 Rheumatology, CHU Hedi Chaker; 3 Higher institute of nursing sciences, Sfax, Tunisia

## Abstract

**Introduction:**

Nurses play a crucial role in patient care. Indeed, this profession requires a high level of emotional, mental and physical workload. Improving the well-being of these workers means taking into account the impact of workload on their job satisfaction.

**Objectives:**

Our study aims to assess the relationship between perceived workload and job satisfaction among nurses.

**Methods:**

We conducted a descriptive, analytical and cross-sectional survey among nurses using a self-administrated questionnaire. We collected socio-professional data. We assessed perceived workload using the National Aeronautics and Space Administration Task Load Index (NASA-TLX). Job satisfaction was evaluated using the single-item measure of job satisfaction.

**Results:**

Our population comprised 202 nurses, 67% of whom were female. The mean age of participants was 35.1 ± 8.1 years. The nurses’ length of service in the department was 6.9 ±7.5 years. The mean score of mental demand, physical demand, performance, effort, frustration level and temporal demand were respectively 77.8±22.6, 76.9±23.5, 67.9±31.9, 81.4±20, 66.6±25.2 and 59.8±30.2. The mean score of Raw TLX was 71.44 ±14.8. Among our participants, 74 (37%) were satisfied with their jobs. We found that the job satisfaction was positively correlated with the overall RAW TLX score (p=0.02) and with the physical (p=0.04) and temporal (p=0.00) demands.

**Conclusions:**

Our findings highlight a correlation between high perceived workload and job dissatisfaction. Therefore, it is crucial to assess and improve working conditions to ensure a safe, comfortable and well-equipped environment, thus promoting nurses’ satisfaction and quality of care.

**Disclosure of Interest:**

None Declared

